# Atomic-Scale Mapping of an Emergent Interfacial Two-Dimensional Electron Gas at Anatase/LaAlO_3_

**DOI:** 10.1021/acs.nanolett.6c01308

**Published:** 2026-10-14

**Authors:** Michael Oberaigner, Manuel Ederer, Sandeep K. Chaluvadi, Pasquale Orgiani, Regina Ciancio, Stefan Löffler, Gerald Kothleitner, Daniel Knez

**Affiliations:** † Institute of Electron Microscopy and Nanoanalysis, Graz University of Technology, Steyrergasse 17, Graz 8010, Austria; ‡ Graz Centre for Electron Microscopy, Steyrergasse 17, Graz 8010, Austria; ¶ University Service Centre for Transmission Electron Microscopy, 27259 TU Wien, Stadionallee 2, Wien 1020, Vienna, Austria; § CNR-IOM Istituto Officina dei Materiali, TASC Laboratory, Area Science Park, Strada statale 14 km 163.5, Trieste 34149, Friuli-Venezia Giulia, Italy; ∥ 18469 Area Science Park, Padriciano 99, Trieste 34149, Friuli-Venezia Giulia, Italy

**Keywords:** Transition metal oxide interface, 2DEG, STEM-EELS, DPC, Oxygen Vacancies, Electronic state mapping

## Abstract

Atomic-level insight into structure, charge distribution, chemistry, and electronic states is essential for understanding emergent properties at oxide interfaces. Here, we leverage the quasi-strain-free anatase TiO_2_/LaAlO_3_ interface as an ideal model system to decouple intrinsic polar discontinuities from complex strain artifacts. By combining atomic-resolution electron energy loss spectroscopy with integrated differential phase contrast, we spatially map the electronic states associated with the formation of a two-dimensional electron gas (2DEG). This correlative approach enables the direct real-space disentanglement of intrinsic 2DEG-related states from extrinsic Ti^3+^ defect states. Supported by density functional theory and inelastic scattering simulations, we reveal a physical superposition: the intrinsic polar electronic reconstruction anchors metallic states directly at the boundary, while oxygen vacancies accumulate further into the film, acting as a donor reservoir. This work not only resolves long-standing ambiguities regarding oxide interfacial conductivity but also provides a robust, transferable framework for related material systems.

## Graphical abstract

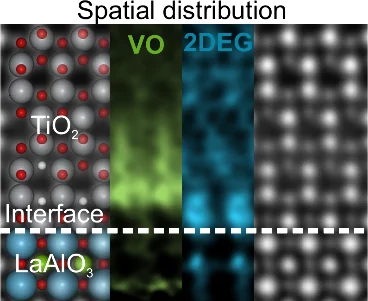

Among the emergent properties reported at transition-metal-oxide (TMO) interfaces, two-dimensional electron gases (2DEG) have attracted particular attention, due to their potential to transform spintronics and oxide electronics.^1–3^ Their distinctive behavior originates from the subtle interplay between lattice structure, electrostatics, and defect chemistry. Accordingly, significant efforts have focused on probing these coupled degrees of freedom to clarify how they jointly govern interfacial conductivity. In practice, however, the strongly localized and interdependent nature of these correlation effects makes it difficult to disentangle individual contributions, and the microscopic mechanisms underlying 2DEG formation remain unresolved.^4^

This lack of knowledge is closely tied to limitations of the experimental techniques currently available to probe these phenomena. While approaches such as angle-resolved photoemission spectroscopy (ARPES), X-ray absorption spectroscopy (XAS), X-ray photoemission spectroscopy (XPS), magnetic/electrical transport measurements and scanning tunnelling microscopy and spectroscopy (XSTM/S)^5–12^ provide valuable insights, they either lack the required spatial resolution or are predominantly sensitive to surfaces rather than buried interfaces. Furthermore, as previously demonstrated for ultrathin anatase TiO_2_/LaAlO_3_ heterostructures,^13^ macroscopic transport measurements are fundamentally limited below a critical thickness of 16 nm, because surface depletion effects completely suppress long-range electrical percolation, rendering such films macroscopically insulating. While a definitive macroscopic demonstration of 2DEG functionality would ultimately require low-temperature quantum transport experiments (such as Shubnikov-de Haas oscillations) on lithographically patterned devices, such measurements are highly challenging here due to these thickness-dependent depletion effects. To bypass this macroscopic percolation bottleneck, the present work employs monochromated STEM-EELS as a highly sensitive local probe of the buried interface. This allows us to directly evaluate the local electronic reconstruction and verify the presence of confined electronic states compatible with the formation of a 2DEG, complementing rather than replacing conventional macroscopic transport studies.

Through this approach, we directly visualize at atomic precision the physical effects associated with the formation of a 2DEG. By correlating the local charge redistribution with the atomic-scale defect landscape, we isolate the fundamental drivers of interfacial conductivity. Our experimental observations are further supported by density functional theory (DFT) simulations and inelastic scattering calculations.

While 2DEGs have been reported at several TMO interfaces, the SrTiO_3_/LaAlO_3_ interface remains the most extensively studied example of nonpolar/polar interfaces.^14^ However, its ∼3% lattice mismatch introduces significant epitaxial strain, causing ionic displacements that convolute strain relaxation with electrostatic responses. The anatase TiO_2_/LaAlO_3_ interface investigated here is closely related yet largely avoids this complication due to a near-perfect structural match (0.1% mismatch).^13,15,16^ For such interfaces, 2DEG formation is often attributed to the polar discontinuity, commonly discussed in terms of electronic reconstruction or polar catastrophe model.^10,14,17^ This quasi-strain-free template allows us to effectively decouple the polar discontinuity from epitaxial strain, ensuring that any observed lattice polarization can be directly attributed to the screening of the interfacial electric field rather than strain artifacts. Furthermore, anatase TiO_2_ offers a crucial spectroscopic advantage: the itinerant 2DEG states (accessible via the O K-edge) can be cleanly separated from localized defect states (probed via the Ti *L*_3_-edge). By leveraging this ideal model system, we bypass the inherent structural and electronic complexities of highly strained interfaces. This enables a correlative approach to isolate the fundamental mechanisms of interface conductivity and directly disentangle the intrinsic 2DEG from extrinsic defect phenomena. Within the framework of the polar catastrophe model, the built-in electrostatic potential is compensated by electron transfer from the valence band of the polar LAO surface layer, rendering the interface conductive. Although widely accepted, the relative importance and microscopic details of additional contributions most notably oxygen vacancies (V_O_) and interfacial chemistry, remain unsettled. Oxygen vacancies are expected to act as *n*-type dopants, have also been linked to electron confinement at the interface, potentially mediated by the interfacial electric field.^18,19^ Another mechanism invokes cation diffusion across the interface: cation intermixing can supply charge carriers that establish a conducting region, or alternatively reduce mobility by acting as scattering centers within the conductive layer.^20–22^

However, the high and largely constant electron mobility observed over a wide range of V_O_ concentrations has been attributed to spatial separation of the mobile charge carriers from the charged oxygen vacancies.^23^ In addition, theoretical considerations suggest that V_O_ formation is energetically unfavorable because a polar discontinuity can induce an energy barrier.^24^

As a result, the role of V_O_ in 2DEG formation and more broadly, the presence and spatial distribution of oxygen vacancies and cations in the interfacial region, remain insufficiently understood. To resolve these ambiguities, we systematically isolate the fundamental mechanisms of interface conductivity. First, we leverage the macroscopically conducting, La-terminated anatase TiO_2_/LaAlO_3_ model system (Figure 1a) as a clean, defect-sensitive baseline. Next, by mapping the coupled structural and electronic responses to the interfacial potential, specifically, the subunit-cell ionic displacements and the resulting charge redistribution, we directly disentangle the intrinsic 2DEG from extrinsic oxygen vacancies in real space. This spatial separation establishes the intrinsic origin of the metallic state.

**Figure 1:**
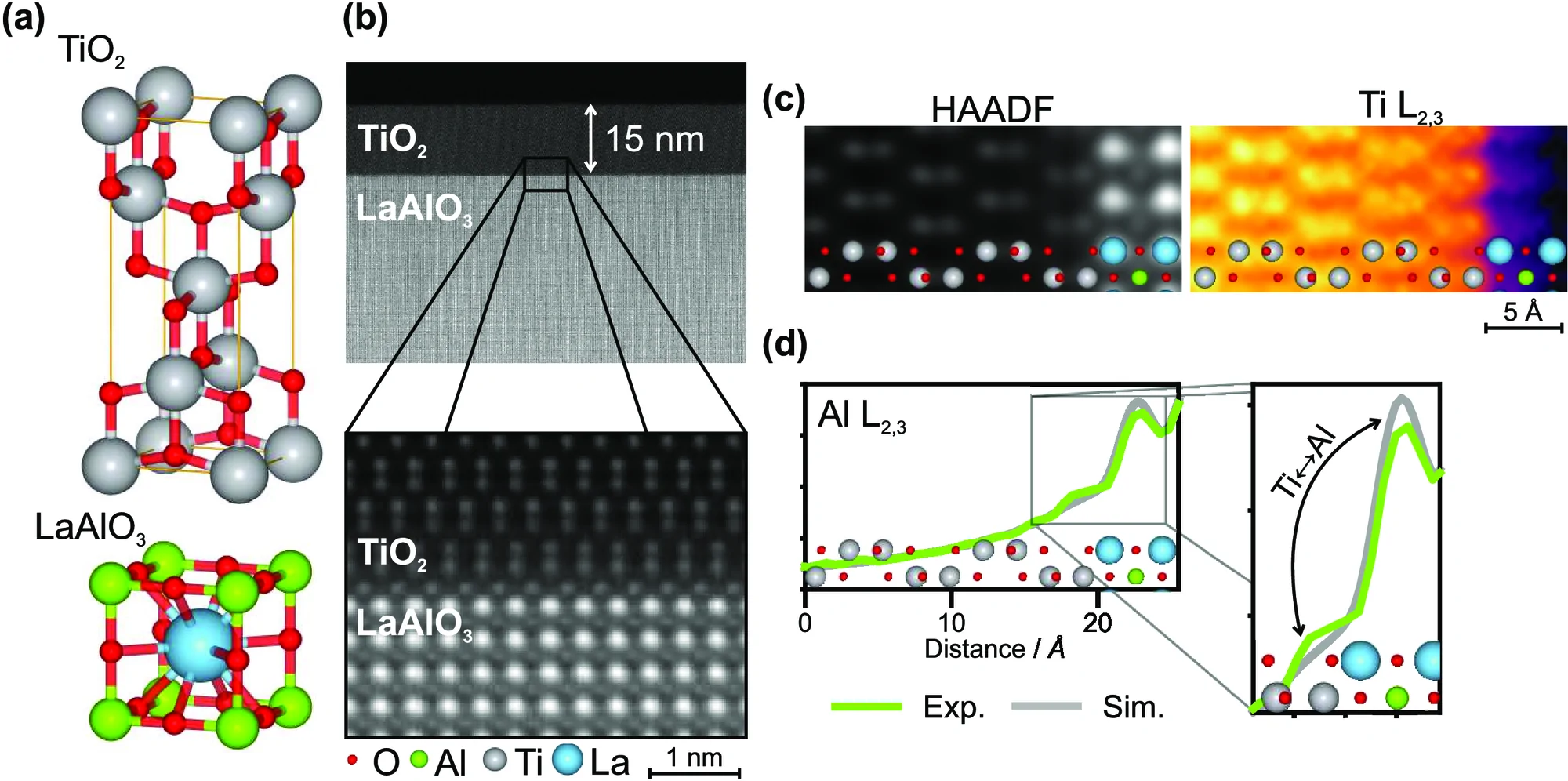
Structural framework and chemical termination of the epitaxial TiO_2_/LaAlO_3_ interface. (a) Unit-cell models defining the expected interface geometry, alongside (b) a cross-sectional HAADF-STEM image confirming high-quality epitaxial growth. (c) Combined HAADF contrast and Ti L_2,3_ EELS mapping isolate the precise termination layer, while (d) the Al L_2,3_-edge profile quantifies the extent of local cation intermixing relative to an idealized abrupt boundary. Ultimately, these initial structural and chemical maps establish that despite minor interdiffusion, the system provides a highly ordered, (LaO)^+^-terminated polar template necessary to drive the subsequent electronic reconstruction.

Anatase TiO_2_ (001) thin films investigated in this work were grown epitaxially on single crystalline LAO (001) by pulsed laser deposition (PLD). Figure 1b shows a representative cross-sectional view of the anatase film and interfacial region, revealing a high-quality, atomically sharp interface, consistent with previous reports.^25,26^ The anatase layer has a uniform thickness of approximately 15 nm. Because of the low lattice mismatch between anatase and LAO, film and substrate are fully matched in plane. The interface can exhibit two principal terminations, which are associated with distinct electronic behavior. In LaAlO_3_, (AlO_2_)^−^ and (LaO)^+^ layers carry alternating formal charges of −1 and +1, respectively, giving rise to an interfacial excess charge. This charge can be compensated for by terraces with different stacking, oxygen vacancy formation, or atomic interdiffusion.^17^ Since a 2DEG forms only for the La-terminated interface (whereas Al-termination results in semiconducting behavior), we focus here on La-terminated interfaces.^13,16,27^ La-termination is identified from the high-angle annular dark-field (HAADF) contrast and further supported by atomically resolved EELS mapping of the Ti L_2,3_ edge signal, as shown in Figure 1c. Despite the presence of cation intermixing between Ti and Al across the terminating layers (Figure 1d), it is not anticipated to affect the 2DEG charge carrier density. This is due to the inefficiency of this species as an *n*-type dopant in TiO_2_.^13^ However, it should be noted that cation intermixing potentially reduces charge carrier mobility.^22^

In order to identify the electronic states associated with the 2DEG, a defect-free interface model was employed for DFT calculation. Resulting calculations of the partial density of states (pDOS) (Figure 2a) predict metallic behavior in the oxygen layers adjacent to the La-terminated interface, manifested by electronic states crossing the Fermi-level. We therefore attribute these interfacial states, which are associated with oxygen atoms, to the electronic states involved in 2DEG formation. To detect the 2DEG related states experimentally, we performed monochromated STEM EELS mapping of the O K edge. Spectra acquired at the interface (yellow rectangle in the corresponding HAADF image, Figure 2b) were compared with those from a region 1.5 nm away (blue rectangle) demonstrating that metallic signatures occur only in the immediate vicinity of the interface. To resolve these weak spectral features with high spatial and energy resolution, we applied a post-processing workflow that identifies and removes artifact-affected data, corrects sample drift, and improves signal-to-noise using multiframe acquisition. The full procedure is described in more detail in the Supporting Information. The resulting O–K edge spectra are summarized in Figure 2c. In the bulk-like region, the spectra show the typical anatase fine-structure, including the crystal-field split t_2g_ and e_g_ peaks; for clarity, only the t_2g_ peak is displayed. In contrast, spectra at the interface exhibit an additional feature at the edge onset near 530.2 eV (highlighted in Figure 2c). Such an onset feature is consistent with the emergence of states at the Fermi level, in agreement with the DFT results. The specimen thickness along the beam direction was determined by PACBED yielding approximately 18 nm. Notably, neither oxygen vacancies nor cation intermixing was included in the structural model used for the electronic-structure and subsequent scattering simulations, indicating that the observed pre-edge signal originates from intrinsic interfacial states rather than VO accumulation or interdiffusion effects. For the multislice simulation, the selected energy was set to 0.4 eV above the Fermi level, whereas the experimental integration window of the experiment was chosen at the corresponding lower energy-loss to minimize contributions from the bulk signal. The resulting map (Figure 2d) shows that the 2DEG related states are confined to a narrow region spanning the first two Ti columns from the interface. The analogous map from STEM-EELS multislice simulation (Figure 2e) based on the pDOS at the defined energy loss agrees well with the experiment, supporting an electronic reconstruction origin for metallic features.

**Figure 2:**
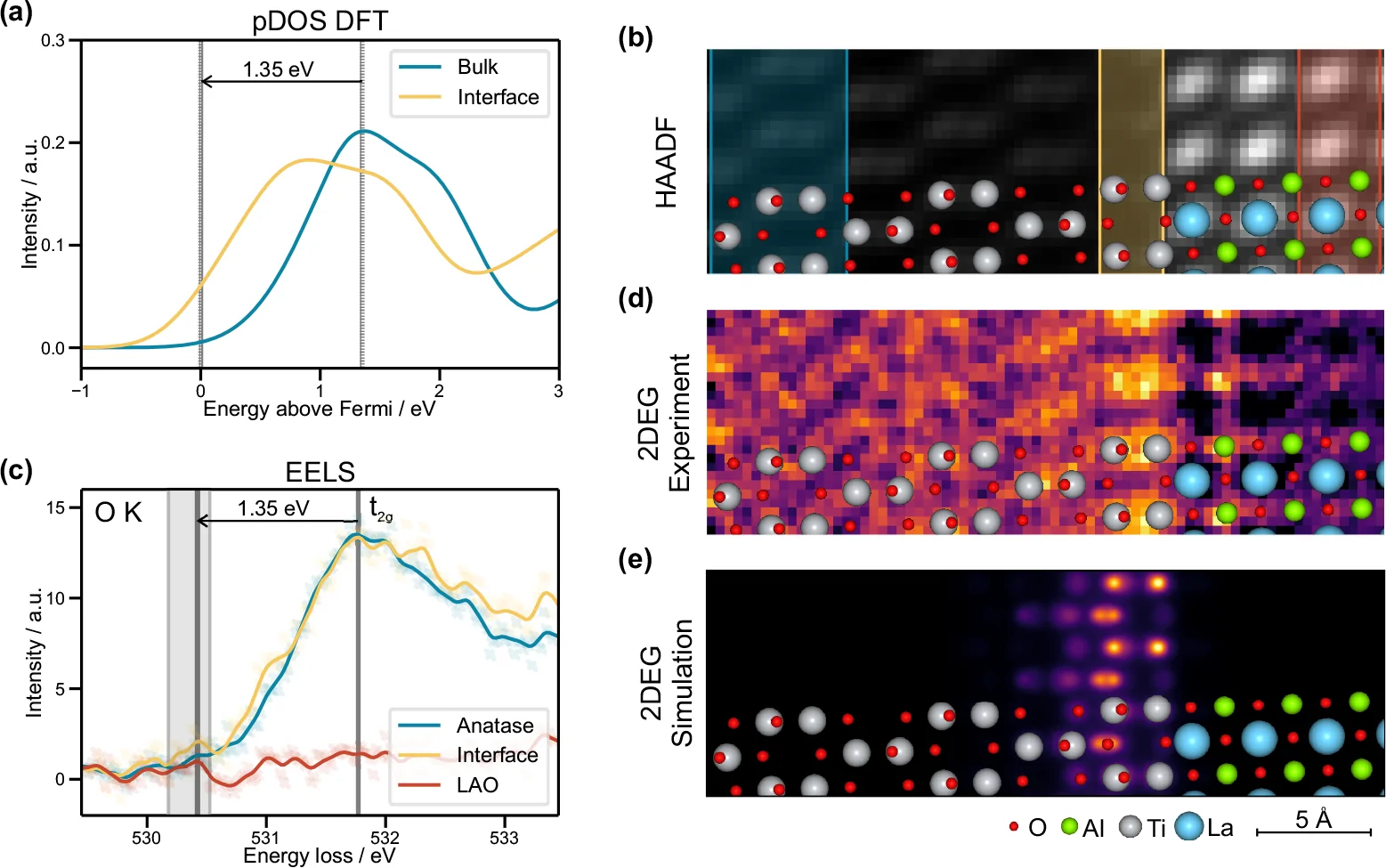
Real-space mapping and spatial confinement of the intrinsic 2DEG. The theoretically predicted metallic states crossing the Fermi level in the calculated oxygen partial density of states (a) manifest directly as a subtle pre-edge onset in the experimental O K-edge EELS spectra (c), extracted from the interfacial and bulk regions marked in the HAADF reference image (b). By integrating this specific pre-edge signature, we obtain the experimental 2DEG real-space map (d), which demonstrates excellent structural agreement with the simulated Fermi-level pDOS distribution (e). This correlative mapping confirms that the metallic states are strictly confined to the first 0.5 nm of the interface, providing direct visual proof of the intrinsic electronic reconstruction.

While the electrons forming the 2DEG belong to a spatially delocalized (itinerant) system, the corresponding EELS signal appears strictly localized on the atomic columns. This occurs because, according to Fermi’s Golden Rule, the EELS transition probability is governed by the spatial overlap between the final states and the highly localized oxygen 1s core-hole initial state. In the following, we use “2DEG states” to refer to these atomically confined signatures detected via the O K-edge pre-edge, which are distinct from extended space-charge regions typically accessed by phase-contrast techniques such as differential phase contrast (DPC) or electron holography. While the multislice approach incorporates both elastic and inelastic scattering, additional effects, including thermal diffuse scattering and higher-order multipole contributions, are neglected or treated approximately to limit computational cost. Consequently, the importance of choosing suitable experimental parameters, such as the collection angle, to minimize the influence of these factors and accurately capture the spatial overlap constraints cannot be overstated (see detailed discussion in Figure S1 in the Supporting Information).

Similar 2DEG-related signatures at the Fermi level are, in principle, also expected at the Ti L_3_ EELS edge. In the present case, the corresponding signal overlaps with spectral contribution from defect-related (Ti^3+^) states,^11^ as shown in Figure S2 in the Supporting Information. Furthermore, multiplet effects, which are not captured by standard DFT calculations, strongly influence the Ti L edge fine-structure, such that the spectra cannot be interpreted as a direct representation of the pDOS (for example, prepeak features at the L_3_ edge).^28,29^ Accurately accounting for these effects would require solving the Bethe-Salpeter equation, which is computationally demanding and beyond the scope of this work. However, it is worth noting that electron–electron interactions can be neglected for the O K edge, because the interaction energy between the O 1s core electron and valence electrons is below 1 meV.^30,31^

To complement this electronic analysis, we directly examined the structural response to the interfacial charge distribution. A resulting local electric field is expected to produce small relative shifts of negatively charged O ions with respect to the positively charged Ti ions. Such atom displacements can be quantified from a high-resolution integrated DPC (iDPC) image of the interface region (Figure 3a), which offers improved contrast and sensitivity for light elements.^32^ Atomic column positions were determined with subpixel precision by fitting two-dimensional Gaussian functions, using the *Atomap* package.^33^ We then measured the out-of-plane distances between neighboring Ti and O columns. The analysis reveals local lattice expansion and compression near to the interface, depending on the orientation of the Ti–O dipoles relative to the field direction with Ti–O separations changing by approximately 10–20 pm. The influence of an interfacial electric field on anatase ion positions is qualitatively reproduced by multislice simulations based on crystal geometries from atomistic calculations (Figure 3b), comparing field-free structure with one subjected to an electric field of 1.5 V/Å. This value was chosen from a set of simulations with varying field strengths by matching the simulated mean Ti–O distance to the experimental values (see the Methods section and Figure S3 in the Supporting Information). However, because the underlying atomic relaxations are obtained using empirical interatomic potentials, we do not assign a quantitative field magnitude and instead conclude that the measured displacements are consistent with a confined interfacial electric field. Interfacial strain and sample tilt can be ruled out as origins of the measured displacements, as shown by additional simulations in the Supporting Information. We therefore attribute the observed displacements to the field associated with the positively charged (LaO)^+^ terminating layer. This field compensates the polar discontinuity between polar LAO and nonpolar TiO_2_, thereby mitigating the polar catastrophe within the electronic framework.^14,34^ Consistent with this interpretation, DPC measurements, based on electron beam deflection, often an indicator of an electric field, agree with the displacements patterns (Figure S6 in the Supporting Information). We note, however, that isolating field-induced contrast from other interfacial contributions, such as changes in diffraction conditions or variations in the mean inner potential that also affect DPC signals, is nontrivial.^35^ Our observations complement earlier work on space charge and electrostatic fields at oxide structural features and interfaces.^36^ Phase-contrast techniques such as DPC and electron holography have been used to map electrostatic potentials in such systems.^37–39^ While interpretation of phase signals can be challenging, these studies emphasize the importance of linking local potential variations to corresponding structural signatures.

**Figure 3:**
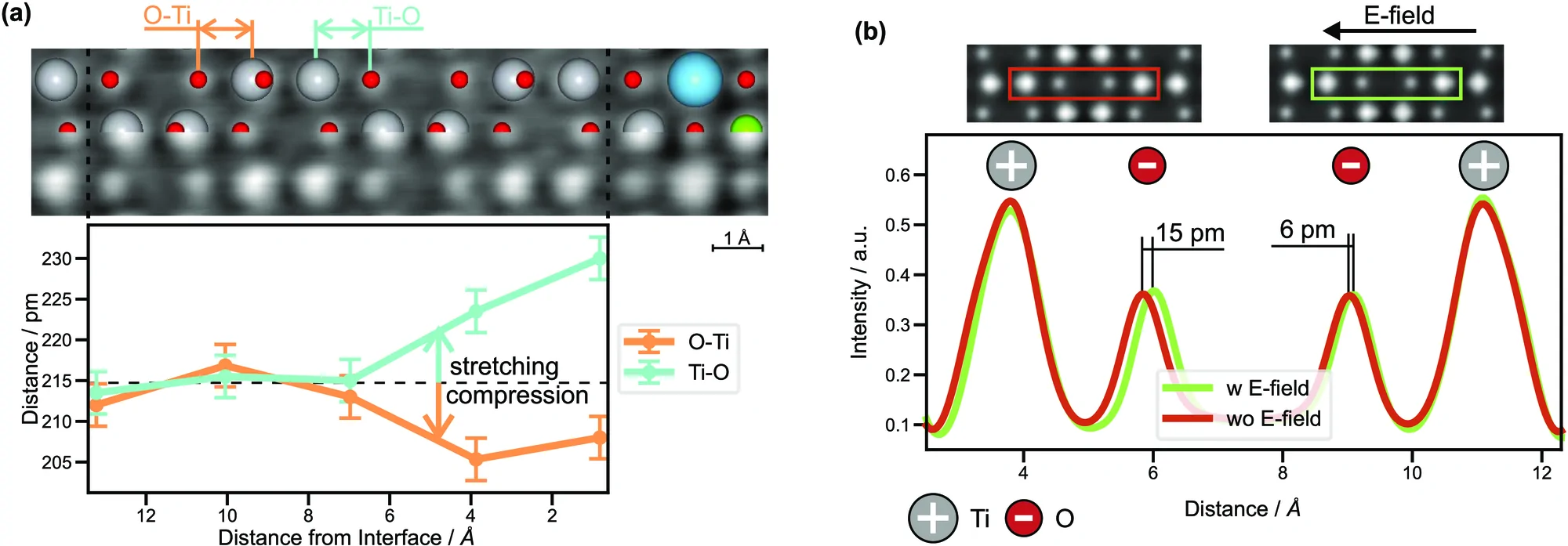
Atomic-scale mapping of the lattice polarization screening the interfacial electric field. High-magnification iDPC imaging of the boundary region (a) reveals a highly directional stretching and compression of Ti–O dipoles, which directly matches the theoretical atomic shifts extracted from our atomistic simulations under an applied electric field (b). This orientation-dependent ionic displacement confirms the presence of a strong, built-in confining potential, physically demonstrating the structural response coupled to the electronic reconstruction.

Taken together, these results support a compensation mechanism dominated by electronic reconstruction, involving charge transfer into interfacial Ti sites to form a confined 2DEG, aided by an interfacial confining electric field. Although we did not observe signatures of lateral atomic reconstruction, such effects have been reported in related polar/nonpolar systems^17^ and may occur alongside the out-of-plane charge redistribution identified here.

Beyond these intrinsic effects, extrinsic defect contributions must also be considered. Oxygen vacancies V_O_ can strongly affect oxide interface properties and are deemed to influence 2DEG formation.^40^ On the one hand, V_O_ act as donor defects, supplying additional electrons and thereby increasing the interfacial carrier density.^23^ On the other hand, they can also induce lattice distortions,^25,41^ which modify the local electronic structure and band alignment at the interface.^42^ To probe spatial variations in the V_O_ concentration, we use the EELS Ti L_3_ e_g_/t_2g_ peak ratio as a sensitive indicator. Variations in these spectral features, often linked to changes in the Ti^3+^/Ti^4+^ ratio,^43^ are directly evident in our EELS data, as shown in Figure 4a, where spectra extracted from the three regions in Figure 4b are compared. The e_g_/t_2g_ ratio map (Figure 4c) reveals an enhanced Ti^3+^ related signal extending into the second and third Ti layers away from the interface, in close agreement with the spatial extent of the confining potential inferred above. In contrast, the terminating Ti layer exhibits an e_g_/t_2g_ ratio comparable to the bulk. Within the polar catastrophe picture, interfacial Ti ions would be expected to approach an average Ti^3.5+^ state due to compensating charge transfer, which would imply an even lower effective oxidation state in the adjacent layers. However, changes in the local O coordination (and the associated crystal-field environment) at the interface also alter the Ti L_3_ fine structure, complicating oxidation state assignment based solely on these features. Finally, the LaAlO_3_ region is masked in the map because fitting in the absence of Ti would introduce artifacts and noise. For this reason, we applied a threshold of 1200 counts in the Ti L-edge as a criterion for including pixels in the Ti L_3_-edge mapping. Together, these structural and electronic signatures confirm that oxygen vacancy accumulation peaks away from the immediate boundary, acting as a crucial setup for isolating their spatial relationship to the 2DEG electronic states.

**Figure 4:**
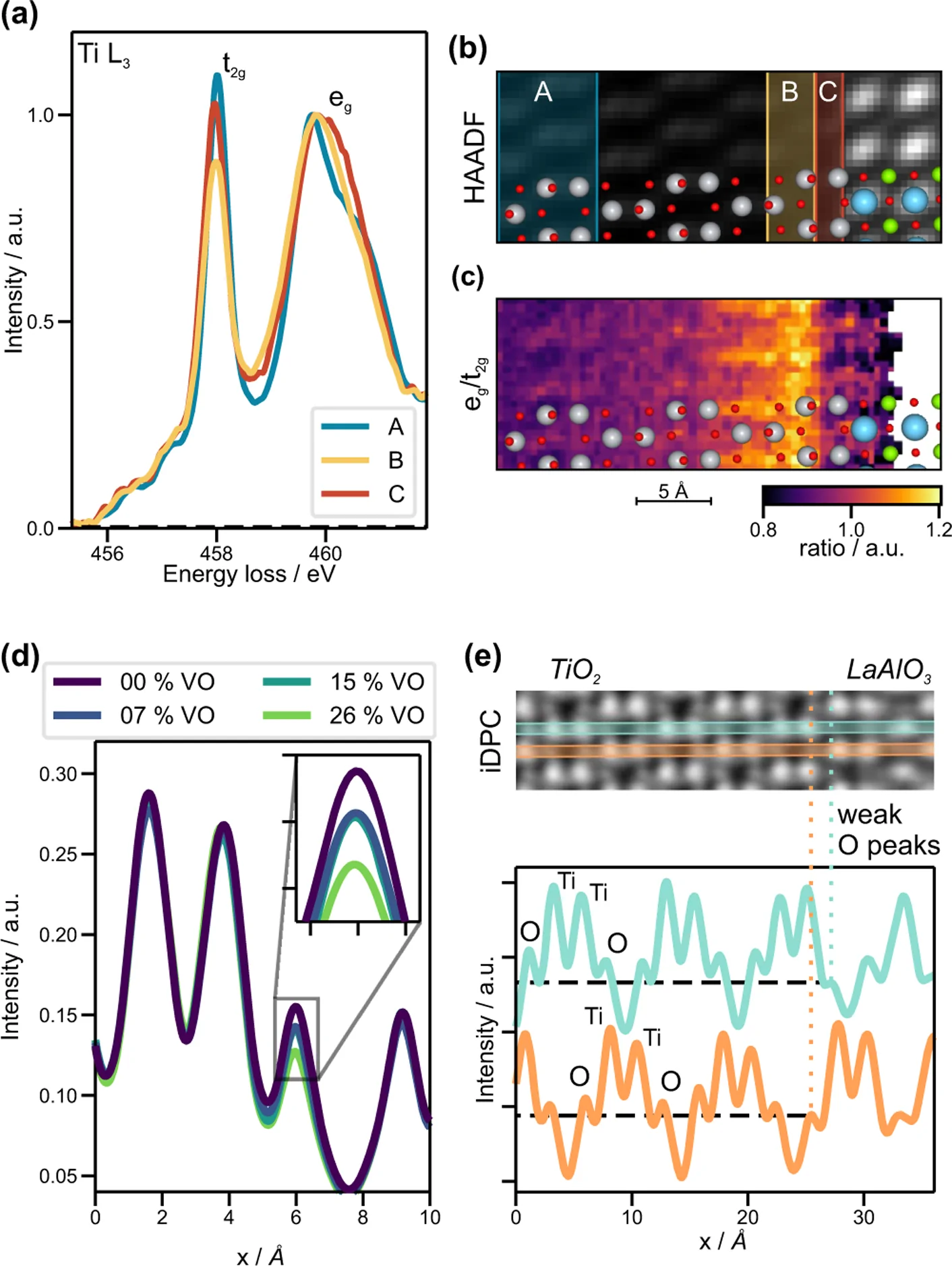
Electronic and structural mapping of interfacial oxygen vacancies. The spatial distribution of Ti^3+^ defect states is electronically mapped via the e_g_/t_2g_ intensity ratio of the Ti L_3_-edge (a, c), extracted from the indicated regions in the HAADF reference image (b). This spectroscopic signature of oxygen vacancies is structurally corroborated by experimental iDPC profiles of the interface (e), which reveal reduced oxygen peak intensities that directly match multislice simulations of vacancy-induced lattice variations (d). Together, these complementary techniques provide a robust, quantitative profile of the extrinsic defect accumulation, revealing that the highest concentration of vacancies peaks distinctly away from the immediate boundary layer.

Additional evidence for an enhanced V_O_ concentration is obtained from the iDPC data (Figure 4d,e). Oxygen columns near the interface exhibit a slightly reduced intensity, consistent with multislice calculations based on defective structures relaxed by Molecular Dynamics.^25^ In contrast, the terminating oxygen layer at the interface shows a higher intensity, in agreement with the EELS results. Notably, the EELS maps of the 2DEG-related states match defect-free simulations, indicating that 2DEG formation is not directly linked to the presence of V_O_. No comparable accumulation of V_O_ is detected on the LAO side (see Figure S7 in the Supporting Information).

Given the limited anatase thickness of approximately 15 nm, surface-related effects such as surface defects or band bending influence the charge distribution at the buried interface. Although the measured 2DEG-related signal and Ti^3+^-associated states are consistent with predominantly intrinsic interfacial behavior, we cannot fully rule out contributions from surface states, particularly following FIB-based sample preparation. Future studies employing thicker films and/or alternative preparation routes should help to better separate surface effects from interfacial phenomena.

The high spatial resolution of our correlative dataset ultimately further allows us to directly disentangle the locations of extrinsic V_O_-related defect states from the O K pre-edge states associated with the 2DEG. Figure 5 provides a comprehensive real-space analysis, comparing normalized intensity profiles extracted from the experimental maps of the O K pre-edge intensity (Figure 2d), the polar ionic displacements (Figure 3a) and the Ti L_3_ peak ratio e_g_/t_2g_ (Figure 4c), which serve as physical proxies for their respective phenomena. From these overlaid profiles, we identify a clear spatial separation between V_O_ accumulation and the 2DEG states: The metallic states are strictly confined to the immediate interfacial region, anchored by the built-in potential, whereas the extrinsic oxygen vacancies predominantly accumulate at distances of approximately 0.5–1 nm from the interface. As reported previously in ref 44, the anatase/LaAlO_3_ interface exhibits an increase in resistivity upon cooling, attributed to Kondo scattering from localized magnetic moments. The high density of V_O_ and the associated Ti^3+^ moments observed near the 2DEG provides a natural source of such moments and it is likely to enhance Kondo scattering at the anatase interface.

**Figure 5:**
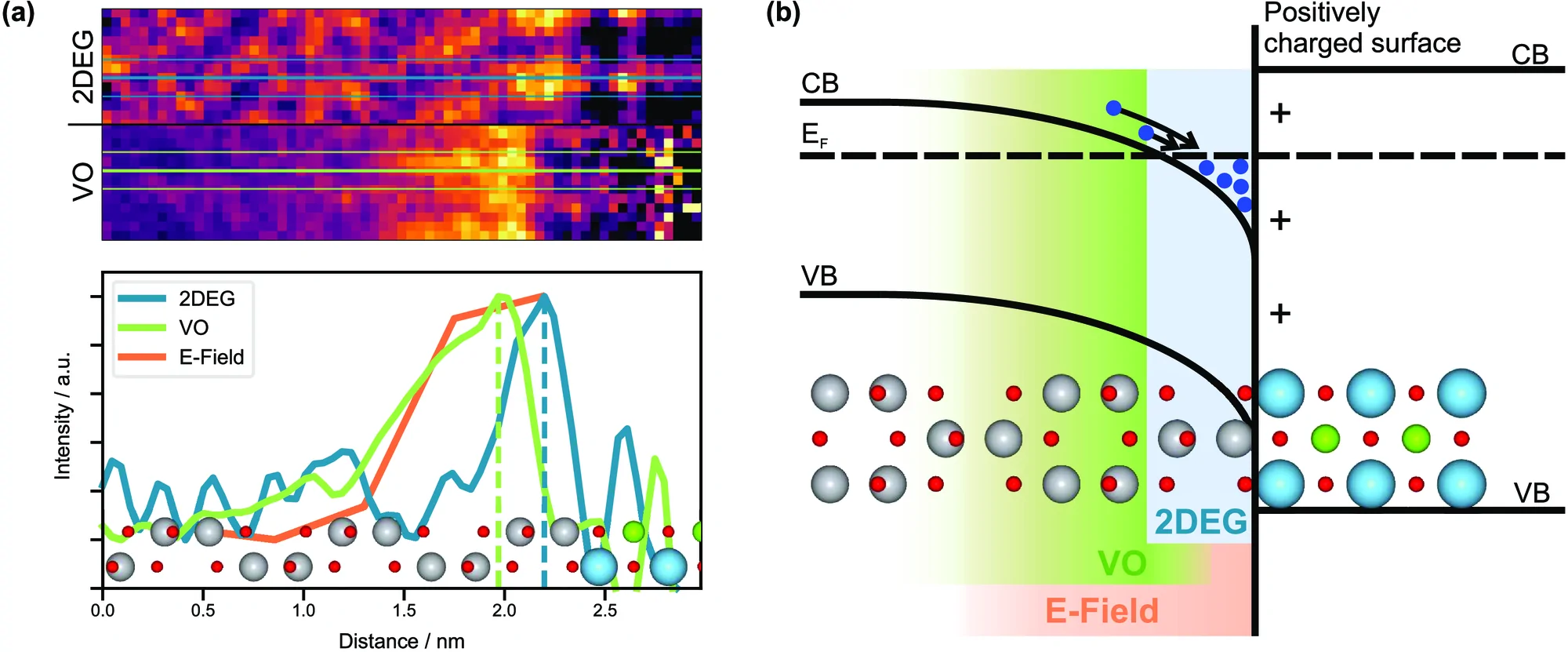
Comprehensive real-space synthesis of the intrinsic 2DEG and interfacial phenomena. By overlaying the normalized intensity profiles of the polar electric field, oxygen vacancy concentration, and 2DEG-associated pre-edge states (a), the distinct spatial extents of each mechanism are directly visualized. This quantitative spatial separation is conceptualized in the corresponding band-bending schematic (b). Ultimately, this combined structural and electronic profile allows us to conclude that the itinerant metallic states are confined by the built-in potential directly at the boundary (0–0.5 nm), remaining physically distinct from the extrinsic defect accumulation.

Despite the simultaneous presence of the positively charged (LaO)^+^ termination and oxygen vacancies, overall charge neutrality is maintained through a combination of electron accumulation directly at the interface and the formation of lower-valence Ti^3+^ deeper within the film. Importantly, our data physically confirm that these compensating delocalized electrons are spatially decoupled from the oxygen vacancies, consistent with an electronic reconstruction driven charge transfer and with Coulomb scattering from charged defects. While the physical vacancies and their localized Ti^3+^ states remain anchored at 0.5–1.0 nm, the itinerant electrons they donate migrate into the confining potential at 0–0.5 nm. Because these donated electrons become delocalized at the interface, they do not contribute to the localized Ti^3+^ defect signature probed by the Ti L-edge, but rather to the itinerant density of states probed by the O K-edge. This spatial disentanglement leads us to the conclusion that the confining potential of the 2DEG is driven by an intrinsic electronic reconstruction, while the spatially separated oxygen vacancies act as a supplementary donor reservoir. This intrinsic physical separation is strongly supported by recent O_2_ dosing experiments,^11^ which demonstrated that vacancy-induced localized states can be selectively suppressed via oxidation while the delocalized, metallic 2DEG response remains intact. Furthermore, this decoupled configuration naturally explains the sustained macroscopic conductivity of the interface, as it is consistent with models of remote Coulomb scattering where the spatial separation of charged defects effectively screens their scattering potential.^23^

Overall, this work elucidates the fundamental microscopic origins of 2DEG formation at oxide interfaces by directly visualizing the governing electrostatic and defect phenomena with unprecedented spatial detail. By leveraging the quasi-strain-free anatase TiO_2_/LaAlO_3_ model system, we successfully disentangle the intrinsic polar electronic reconstruction from extrinsic defect accumulation. Our correlative approach reveals that while the confining potential and the itinerant metallic states are anchored strictly at the boundary by the built-in electric field, supplementary oxygen vacancy donors accumulate further into the film. Establishing this spatial and electronic disentanglement is paramount for understanding 2DEG transport and for rationally engineering functional TMO interfaces. Moving beyond the ambiguity of highly strained systems, our findings confirm that the interface acts analogously to a modulation-doped quantum well, allowing for the deliberate physical decoupling of intrinsic charge carrier generation from extrinsic defect-scattering centers. Ultimately, this framework not only resolves long-standing questions regarding interfacial conductivity but also provides a robust physical foundation for the rational quantum engineering of related complex oxide heterostructures, such as LaAlO_3_/LaVO_3_ ^45^ and SrVO_3_/SrTiO_3_,^46^ where tailoring the interplay between intrinsic polar fields and extrinsic defects is essential for next-generation functional devices. Furthermore, the novel correlative EELS and iDPC methodology established here provides a highly transferable framework for directly imaging these emergent phenomena at the atomic scale.

## Supplementary Material



## Notes

Additional experimental and simulation details, discussion of EELS acquisition parameters, as well as details on methods and data processing steps (PDF)
