# Supplementary material for: Atomic-Scale Mapping of an Emergent Interfacial Two-Dimensional Electron Gas at Anatase/LaAlO3
Source: Nano Lett. 2026 Oct 14;26(42):14507–14. doi: 10.1021/acs.nanolett.6c01308 (PMC13721177; doi:10.1021/acs.nanolett.6c01308)
Supplement: Supplementary file 1 [file nl6c01308_si_001.pdf]

Supplementary Information: Atomic-Scale Mapping of an Emergent Interfacial Two-Dimensional Electron Gas at Anatase/$LaAlO_3$

Michael Oberaigner,[†,‡] Manuel Ederer,[¶] Sandeep K. Chaluvadi,[§] Pasquale Orgiani,[§] Regina Ciancio,[§,‖] Stefan Löffler,[¶] Gerald Kothleitner,[*,†,‡] and Daniel Knez[*,†]

†*Institute of Electron Microscopy and Nanoanalysis, Graz University of Technology, Steyrergasse 17, Graz, 8010, Austria*

‡*Graz Centre for Electron Microscopy, Steyrergasse 17, Graz, 8010, Austria*

¶*University Service Centre for Transmission Electron Microscopy, TU Wien, Stadionallee 2, Vienna, 1020, Austria*

§*CNR-IOM Istituto Officina dei Materiali, TASC Laboratory, Area Science Park, Strada statale 14 km 163.5, Trieste, 34149, Italy*

‖*Area Science Park, Padriciano 99, Trieste, 34149, Italy*

E-mail: gerald.kothleitner@felmi-zfe.at; knez@tugraz.at

The present document provides additional information on the analysis presented in the main manuscript and addresses the following aspects:

- Details regarding the applied experimental and computational methods
- Effect of the collection angle on the 2DEG mapping

- Analysis of the Ti $L_{2,3}$ fine structure
- Simulated impact of the electric field, mistilt, and strain on atomic positions in anatase
- Experimental DPC image of the $TiO_2/LaAlO_3$ interface
- Investigation of cation intermixing at the interface and V_O in $LaAlO_3$
- Details about the data post-processing

Experimental and computational Methods

Sample preparation

The thin layer of anatase was epitaxially grown on (001)-oriented $LaAlO_3$ substrates by pulsed laser deposition (PLD) using a KrF excimer pulsed laser source with a wavelength of 248 nm. A laser energy density of about $2\,J\,cm^{-2}$ and a laser repetition rate of 1 Hz were used. The growth temperature was set to 700 °C and the target-substrate distance was about 5 cm. A stoichiometric single-crystalline rutile TiO_2 target was used and the oxygen pressure was approximately 10^{-4} mbar during growth. The TEM sample was prepared by focused ion beam (FIB) milling at 30 keV and 5 keV, utilizing a *Nova 200 NanoLab* from *FEI*. The FIB lamella was polished by low-voltage Ar^+ ion milling at 900 eV utilizing a *Fischione Nanomill*. Before STEM investigations, the specimens were baked at 190 °C for 2 h in vacuum and cleaned for 10 s with an Ar/O plasma to reduce contamination.

STEM-EELS and DPC

The experiments were performed on an *FEI Titan[3] G2 60-300* equipped with a monochromator and a C_s probe corrector. The EELS signal was recorded by a direct electron detection camera, *K2* by *Gatan Inc.* A HAADF image was recorded simultaneously with each spectrum image, which was used as a reference image for multi-frame spectrum summation and

spatial alignments during post-processing. The images were taken at 300 keV with an EELS semi-collection angle of 100 mrad and a beam semi-convergence angle of 15.5 mrad. The beam current was approximately 40 pA with an energy resolution of 250 meV (full width at half maximum of the zero-loss peak) with the Wien filter. For the DPC measurements, the microscope was operated without the Wien filter. The semi-convergence angle was varied between 15.6 mrad and 19.6 mrad. The signal was recorded by a segmented annular dark-field detector, which is divided into four quadrants with a collection angle of 6-34 mrad. The specimen thickness values, which were used for corresponding simulations, were determined by employing the automated PACBED analysis based on pre-trained convolutional neural networks, as described elsewhere in more detail[1,2].

Data post-processing

STEM-EELS data were denoised by a combination of a multi-frame integration approach and multivariate analysis including automatic control mechanisms assessing data quality, such as clustering the SI and rating frames in the multi-frame approach by an ℓ_2-norm. The procedure is described in detail in the *Post-processing sequence* section.

To map the Ti^{3+}/Ti^{4+} ratio, Voigt profiles, which are a convolution between a Lorentzian and Gaussian function, were fitted to the e_g and t_{2g} peaks from the Ti L_3 edge. These profiles consider the Lorentzian component, which is caused by the lifetime broadening, and the Gaussian part, which is introduced by the energy spread of the primary electrons. The peak width and the shape factor were determined by fitting the spectrum of the summed SI, and these parameters were locked for the amplitude fitting at each pixel afterwards.

DFT calculation

All *ab initio* density functional theory calculations were performed using the all-electron, full-potential augmented plane wave method as implemented in WIEN2k[3]. For the exchange and correlation potential, the PBE-GGA[4] was chosen. The number of plane waves was limited

to 4818 plus 428 local orbitals. The full Brillouin zone was divided into a $13 \times 13 \times 1$ **k**-point mesh. For the atomic structure, we constructed a supercell consisting of 2.5 anatase unit cells and 4.5 LAO unit cells. The periodic supercell incorporates a La–Ti terminated interface and an Al–Ti terminated interface, although only the former is of significance for this work.

Image simulation

The STEM-EELS images were calculated using inelastic multislice simulations[5]. The code incorporates a standard multislice algorithm[6] which simulates the elastic electron propagation through the sample. For the inelastic interaction between the probe beam and the sample electrons, the mixed dynamic form factor (MDFF)[7] was calculated. The MDFF includes the atomic-site-dependent cross density of states and the radial wave functions of the one-electron Kohn-Sham orbitals of the corresponding electronic transition. Both were extracted from the DFT calculation described in the previous section.

Molecular dynamics

Energy minimization of anatase supercells (15x15x8 unit cells) was performed with a varied external applied electric field, between 0 V/Å and 3 V/Å along the [001] crystallographic direction. The simulations were performed with the Large-scale Atomic/Molecular Massively Parallel Simulator (LAMMPS)[8] using a modified embedded atom method potential[9] and conjugate gradient minimization. Defective anatase geometries were equilibrated using a ReaxFF potential with parameters optimized for V_O[10]. Energy minimization was achieved by applying a conjugate gradient minimization with cell size optimization, followed by a damped dynamics step, following procedures developed in our previous work.[11]

Interface

Spectroscopic Signatures and choice of experimental EELS parameters

Supplementary Fig. 1 demonstrates the importance of selecting suitable experimental parameters. The same experiment as in Fig. 2 of the main text was performed, mapping the 2DEG with the O K-edge. However, a smaller collection angle of 24 mrad was used instead of 100 mrad. While the experiment with 100 mrad matches the simulation, the atomic contrast at 24 mrad differs from the simulation. This is likely due to thermal diffuse scattering not being considered and, more importantly, higher-order multipole terms being neglected.[12] As a general rule, increasing the collection angle suppresses these pronounced atomic features, making comparisons with simulations more reliable.

However, this methodological choice entails a critical spectroscopic trade-off governed by the angular dependence of the inelastic scattering cross-section. We are monitoring the O K-edge, a core-loss transition where the initial 1s state is highly confined. Consequently, both the localized Ti^{3+} defect states and the itinerant 2DEG states exhibit transition charge densities that are tightly bound to the atomic site, yielding broad momentum distributions in q-space. The varying visibility of these states at different collection angles is therefore a consequence of signal-to-background optimization.[13]

To understand this difference in visibility, one must consider the spatial overlap governing the transition probabilities. The Ti L-edge probes transitions into highly localized Ti^{3+} defect states, resulting in a massive spatial overlap and an intense spectral peak. In contrast, the O K-edge pre-edge region probes transitions into the itinerant 2DEG. Because this final state is spatially extended across the interface, its local amplitude at any single oxygen site is inherently lower than that of a strongly localized defect state. This mathematically results in a subtle onset rather than an intense, sharp peak, meaning the 2DEG states possess an inherently weak scattering cross-section compared to the prominent defect states. Conse-

quently, the visibility of this weak signature is highly sensitive to the signal-to-background ratio (SBR), which is dictated by the EELS collection angle. At the larger collection angle (100 mrad) used in the main text, the aperture integrates over a wide momentum range. While this successfully captures the intensity of robust localized defects, it simultaneously integrates a vast amount of high-q inelastic background, such as multiple scattering and non-dipole transitions. This massive background accumulation severely degrades the SBR, effectively drowning out the subtle 2DEG signature. Therefore, reducing the collection angle to 24 mrad (as shown here) acts as a spatial filter. By strictly isolating the low-q, forward-scattered regime where dipole transitions peak and the high-angle background is rejected, we optimize the contrast of the edge onset. While this low-angle regime preserves on-axis elastic channeling artifacts, complicating visual comparison with our multislice simulations, it successfully recovers the theoretically predicted Fermi-level step, directly confirming the itinerant nature of the 2DEG.

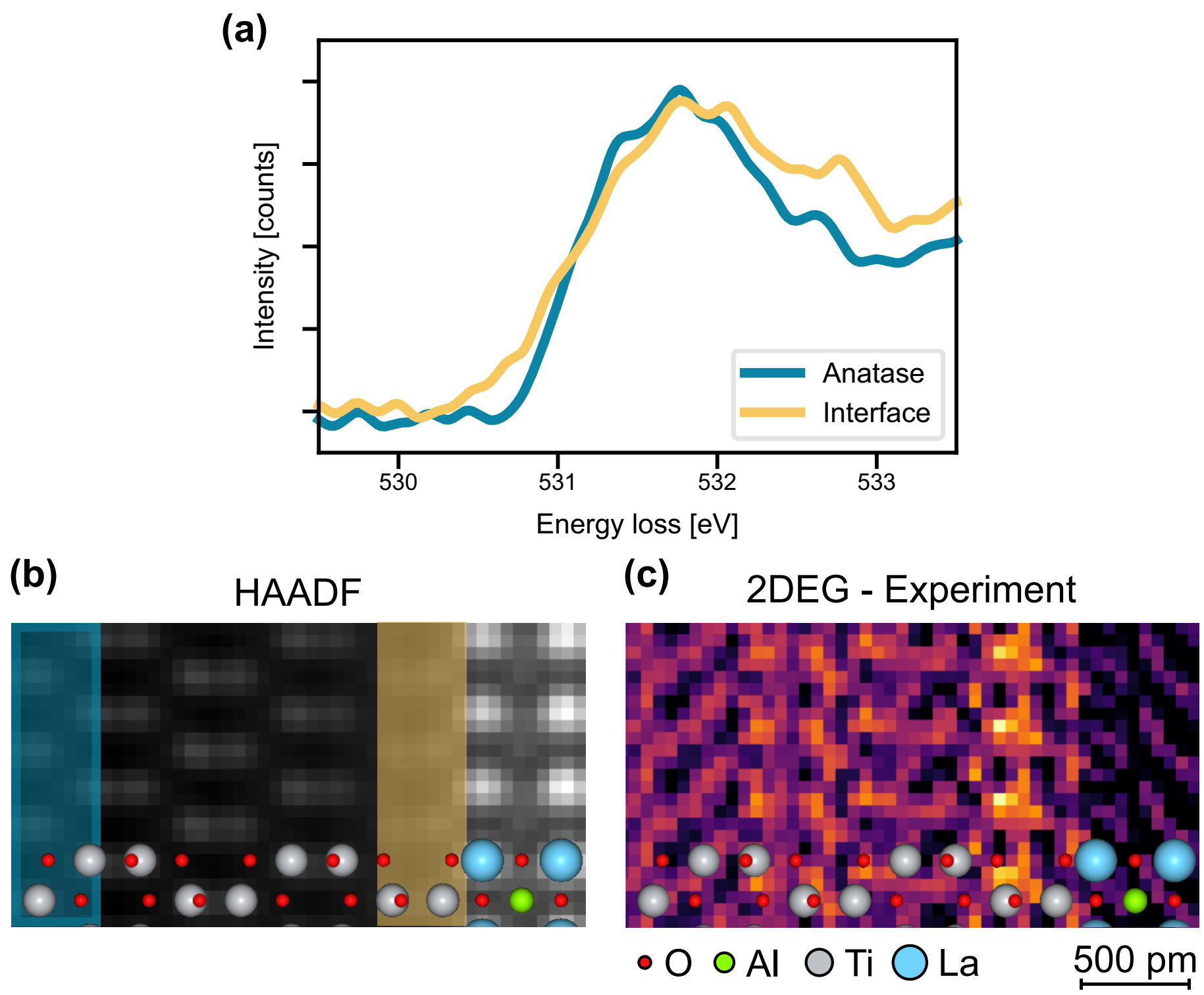

Supplementary Fig. 1: 2DEG mapping with the O K edge with a 24 mrad collection angle. (a) O K-edge comparison between bulk and interface. (b) HAADF image corresponding to the 2DEG map in (c).

Fine structure mapping by Ti ELNES

The interface can be identified as La-terminated by integrating the Ti L edges (Supplementary Fig. 2a). As indicated in Ref.[14], the Ti L_3 edge of Ti^{3+} rises earlier than for Ti^{4+}. A small integration window at the onset of the Ti L_3 edge, which is associated with Ti^{3+}, is used to image the distribution of these defects (2b). This can be localized to the second and third Ti layers in front of the termination (2c).

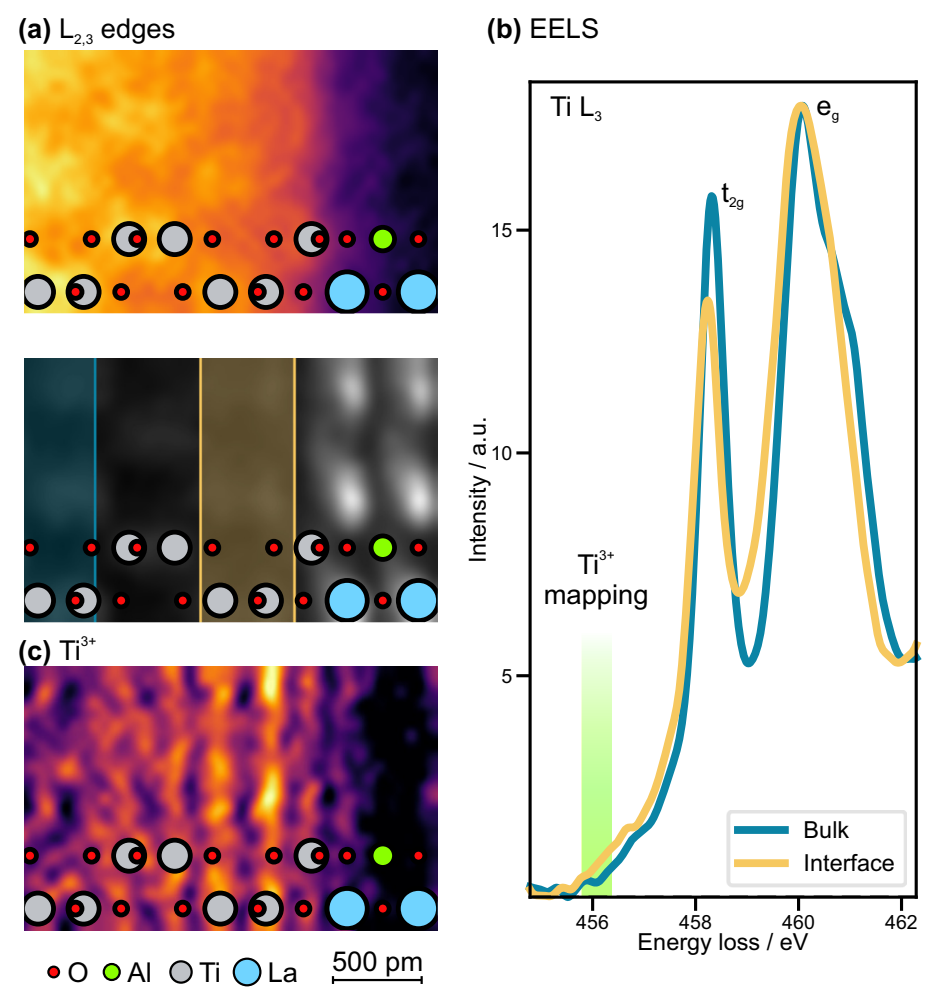

Supplementary Fig. 2: Fine structure mapping with the Ti L_3 edge. (a) Mapping of the $L_{2,3}$ edge demonstrating La-termination with corresponding HAADF image. (b) EELS-Spectrum comparison of the Ti L_3 edge between bulk and interface with the marked integration region to generate the map of Ti^{3+} in (c).

Simulation of atomic displacements with applied electric field

Supplementary Fig.3 displays the distances between the Ti-O and O-Ti dipoles, as obtained from energy minimization with empirical potentials, as a function of simulated electric fields. By comparing these distances with experimentally measured values, we determine a value of 1.5 V/Å as a rough estimate for the field strength within the interface region.

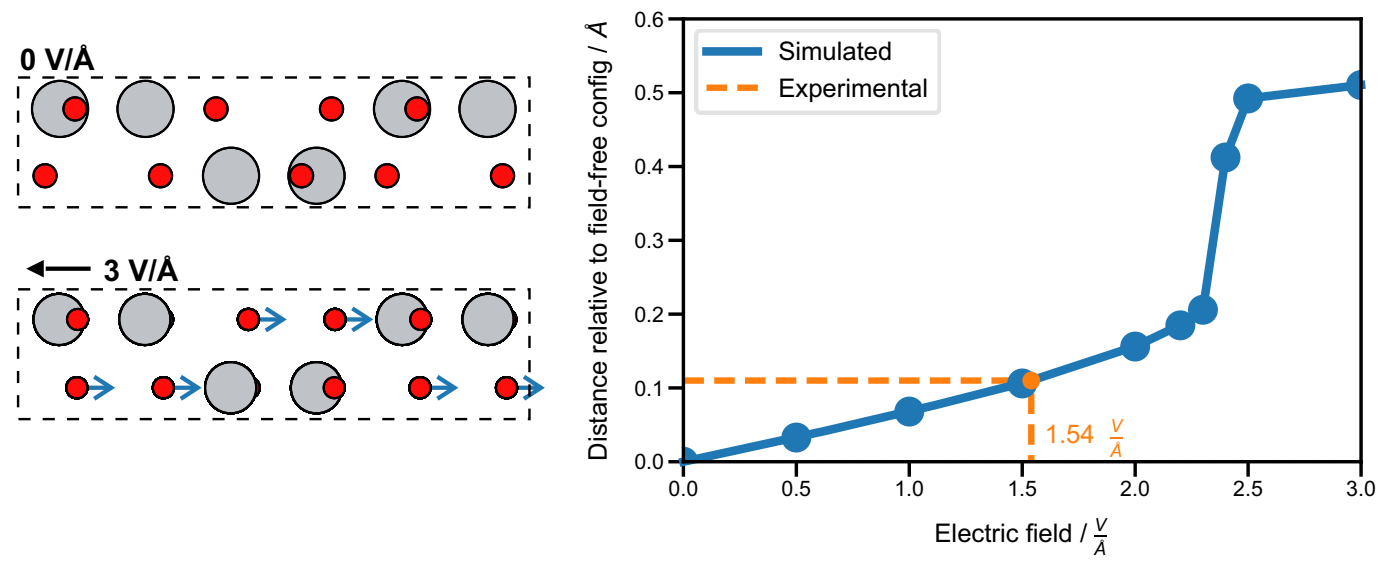

Supplementary Fig. 3: Averaged distances relative to the field-free configuration between Ti and O taken from simulations with varied electric field applied parallel to the [001] crystallographic orientation. The dotted lines mark the measured distance from the experimental iDPC image.

Simulation of atomic displacements by strain and defects

The effects of atomic displacement induced by strain and defects on the distances measured from the iDPC images were investigated by performing additional multislice simulations based on atomistic structures obtained from MD. Due to the complexity of force fields between LAO and anatase, we simplify our model interface by using only anatase and adjusting the atomic position of Ti and O at one side to approximate the interface mismatch. These positions are then fixed and the rest of the supercell is relaxed. Subsequently, these configurations are used for DPC simulations. Because the observed effects were very small for a strain value of 0.1 %, we also conducted simulations with a higher value of 1 % to make strain effects better visible (Supplementary Fig. 4a). Furthermore, a case with 0.1 % mismatch and 15 % oxygen vacancies at the interface (Supplementary Fig. 4b) is included. From our simulations, we can conclude that, although rather high values for strain and oxygen deficiency are chosen, the ionic displacements in the obtained models are still well below the experimentally determined values. We further see that the changes in the displacements are mostly independent of the orientation of the Ti-O columns with regard to the interface, in contrast to our observations, thereby providing further evidence that the observed displacements are indeed caused by an electric field.

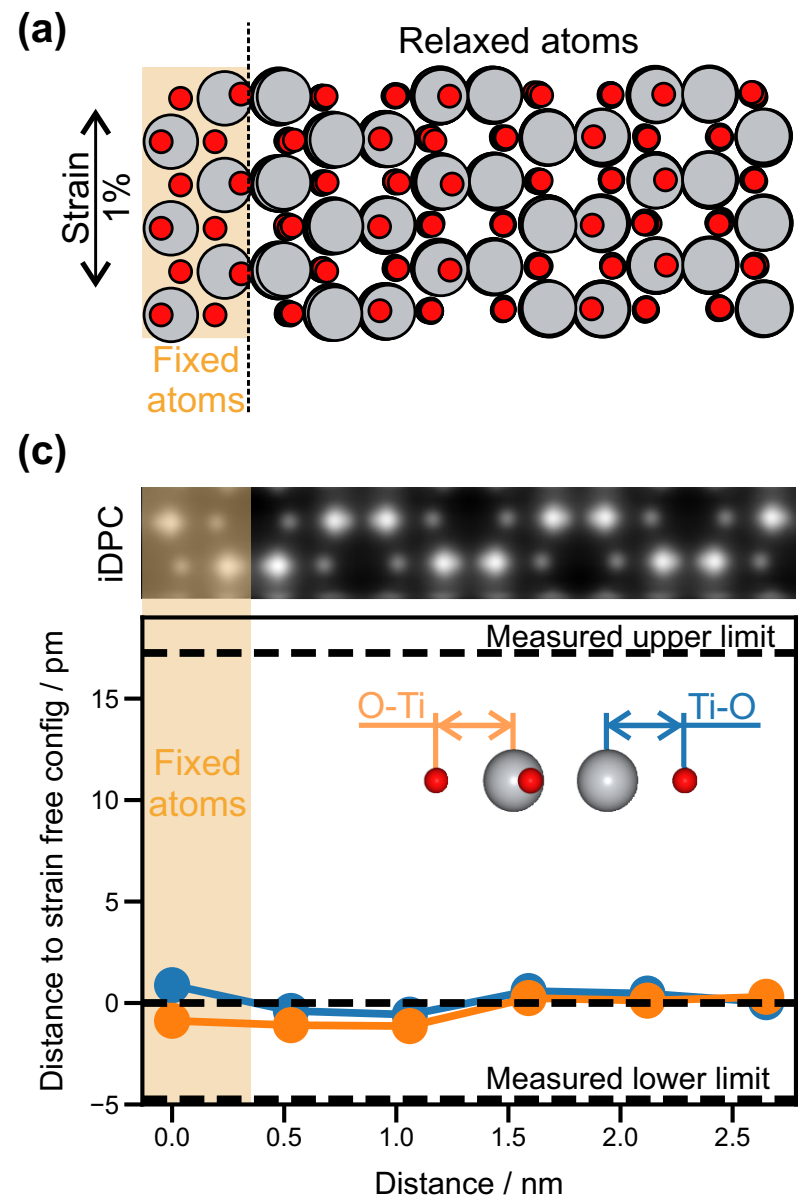

Supplementary Fig. 4: The effect of strain and oxygen vacancies on the atomic positions in anatase. (a, b) Section of the supercell used for MD simulations. (c, d) Length change of the dipoles across the cell depending on their orientation.

Simulation of atomic displacements by mistilt

It is known that a specimen mistilt with respect to the electron beam can also result in a shift of atomic positions in the resulting images. The impact of this phenomenon is more pronounced for iDPC imaging as compared to HAADF[15]. In order to exclude that the observed ionic displacements are caused by such slight mistilts, a tilt-series of iDPC simulations was performed and the obtained atom positions were fitted with *atomap* as in the experiments. Supplementary Fig. 5a provides the distances between the Ti and O columns at varying thicknesses and mistilt angles. It can be seen that the experimentally observed behavior cannot be explained by a mistilt, as the variations are either too small or shifting in the same direction. The mistilt has a negligible effect on the atomic positions at the estimated thickness. The thickness is approximately 5 nm, as evidenced by the comparison of the experimental and simulated iDPC images (Supplementary Fig. 5b).

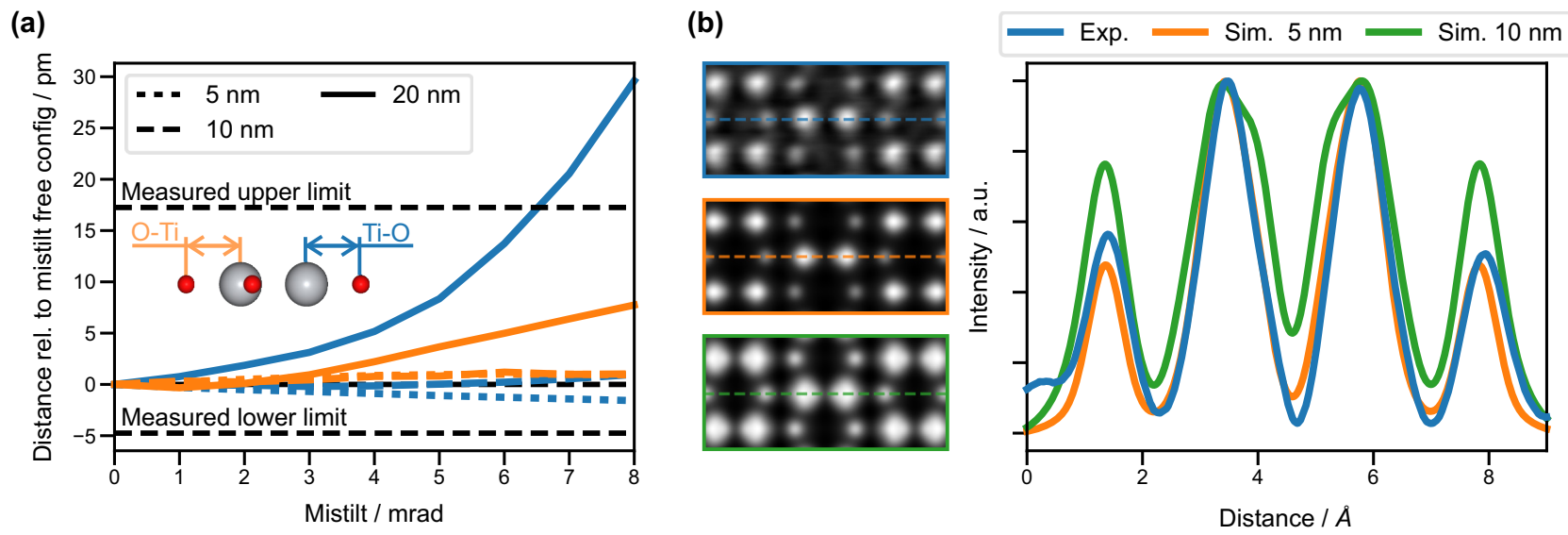

Supplementary Fig. 5: Simulated iDPC tilt-series. (a) Distances between Ti and O at different specimen mistilts and thicknesses. (b) Comparison between simulation and experiment iDPC images.

DPC Images

Supplementary Fig. 6 shows the observed electron beam deflection at the interface, which matches the displacement patterns described in the main text. However, other interfacial contributions can also impact electron beam deflection.

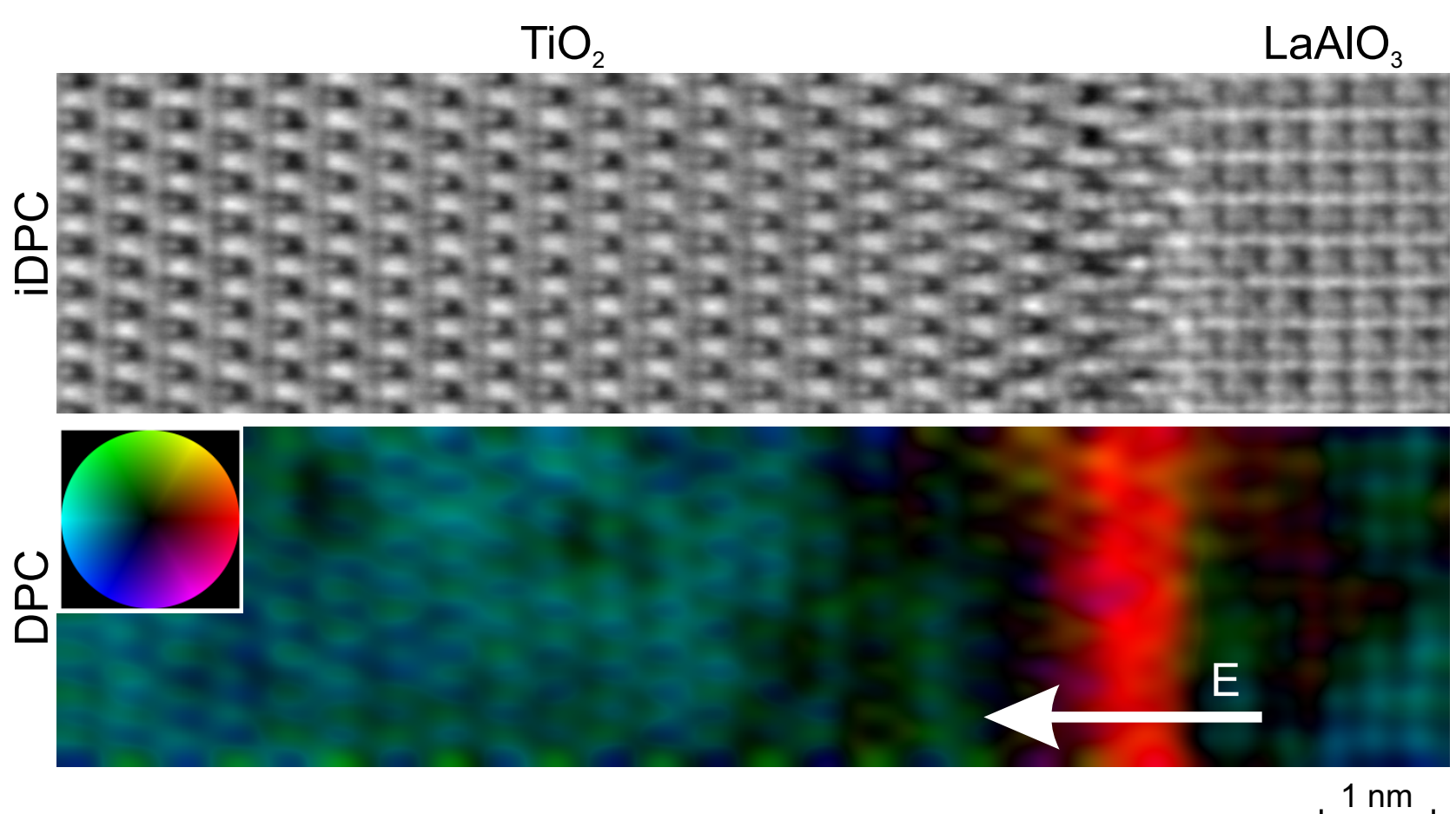

Supplementary Fig. 6: DPC image of the $TiO_2/LaAlO_3$ interface.

Oxygen vacancies in $LaAlO_3$

Similar to the oxygen vacancy detection in TiO_2, we also searched for defects in $LaAlO_3$. Since the L-edges of La have a very high energy loss at around 6000 eV, the M-edges are investigated. In Supplementary Fig. 7a, the edges of the interface and the bulk are compared. Thereby, neither a shift nor a ratio change of the edges can be observed. Furthermore, the

differentiated DPC (dDPC) image does not show any significant changes indicating oxygen defects (Supplementary Fig. 7b). Therefore, an accumulation of oxygen vacancies, as in TiO_2, can be excluded.

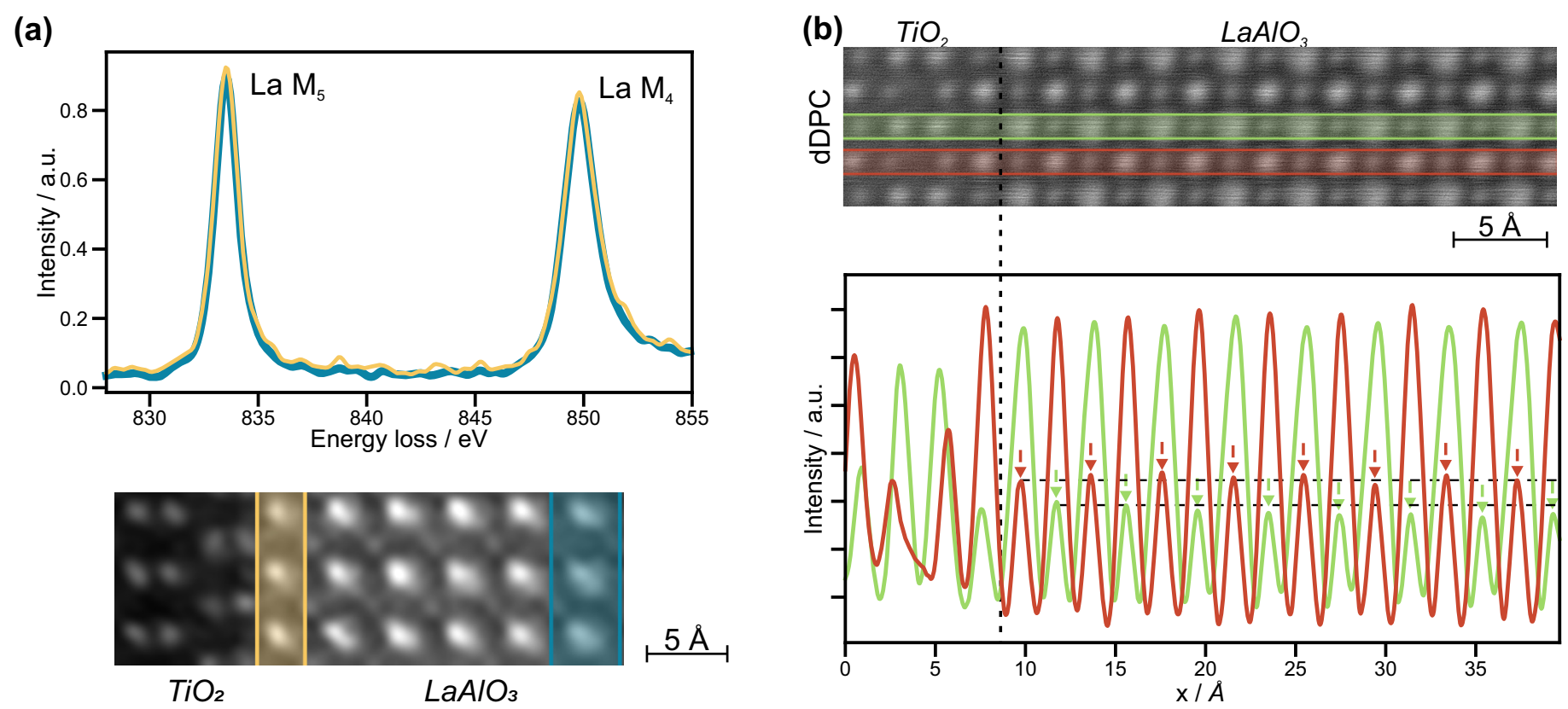

Supplementary Fig. 7: Experiments to detect oxygen vacancies in the $LaAlO_3$ towards the interface. (a) Comparison of the M-edges of La in the bulk and at the interface. (b) dDPC image of the interface with corresponding profiles.

Post-processing sequence

Averaging a multi-frame spectrum image without considering that EELS spectra in different areas can have slightly different features will cause mixing of the signals and ultimately smear out features that are important for mapping orbitals. Therefore, the spectrum image is clustered to detect and visualize changes in the EELS spectra, which cannot be seen in the dark-field image. For this purpose, an algorithm suggested by[16] for unsupervised classification of EELS spectra is implemented. In short, the dimension and the noise of the spectrum image are first reduced by the efficient PCA algorithm. To account for the shot-noise-dominated spectra from the direct detection camera, we use a weighted PCA (wPCA).[17] The dimension is further decreased with the non-linear dimensionality reduction method t-SNE (t-distributed Stochastic Neighbor Embedding).[18] The signal is then clustered using the unsupervised OPTICS (Ordering Points To Identify the Clustering Structure) algorithm, which does not require *a priori* knowledge of the number of clusters, making it possible to

detect unknown clusters.[19] By clustering the spectrum image, slight changes caused by various influences can be visualized, such as decreasing beam current (Supplementary Fig. 8). These regions can be excluded from further processing.

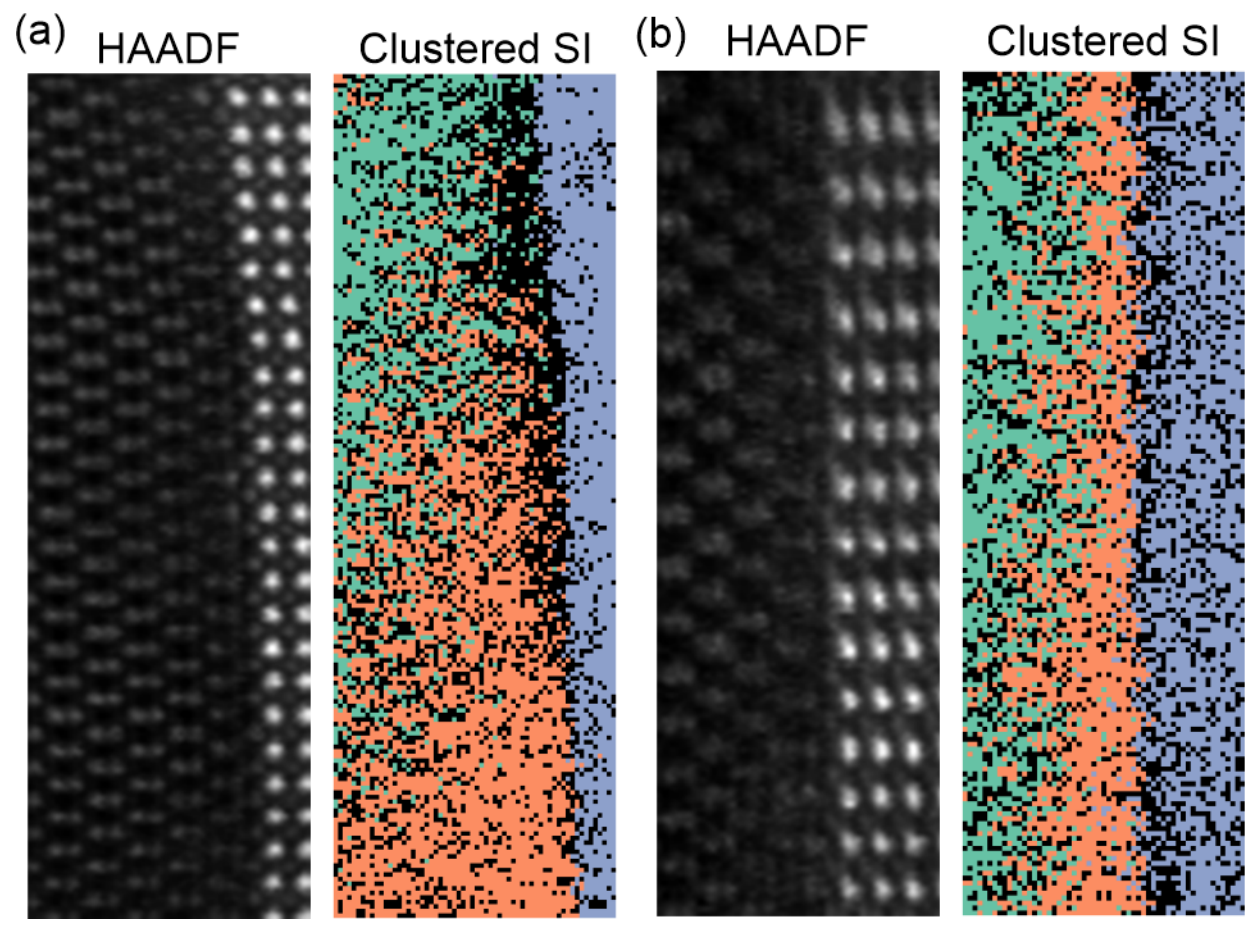

Supplementary Fig. 8: Clustering algorithm on two different spectrum images. (a) Non-uniform interface, due to decreasing beam current along the interface. (b) Uniform interface. For a more detailed analysis, the averaged spectra of each cluster can be investigated.

In order to further minimize artifacts due to the alignment and averaging procedure of the single SI patches, the linear specimen drift is corrected. For this purpose, the exact atom positions in the region of interest are determined by the dark-field image, utilizing the *Python* library *Atomap* (Supplementary Fig. 9a).[20] With the knowledge of the theoretical crystal structure and the determined atom positions, the averaged lattice vectors are transformed to the theoretical lattice vectors.[21] This rigid transformation is applied to the dark-field image and the spectrum image. For cropping smaller frames from the original image, the determined atom positions of the same species are used as the center for each frame (Supplementary Fig. 9b). These frames are stacked and aligned by a rigid registration based on cross-correlation and non-rigid registration based on a gradient descent technique to align scan distortions (Supplementary Fig. 9c).[22] A maximum shifting length at the rigid registration is set to avoid jumping to the next atomic column. This is critical, for example, when mapping the titanium e_g states in rutile, because the atoms exhibit nearly no differences in the HAADF reference image, but the shape of the e_g state is rotated 90° to each

other.[23]

Depending on the quality and sampling rate of the dark-field image, the aligning algorithm can fail or the quality of the slice itself can be insufficient, which would degrade the spatial resolution of the averaged image. Therefore, an ℓ_2-norm is introduced to automatically detect and remove such faulty slices from the stack. The norm is calculated pixel-wise between each normalized frame and the normalized averaged image. The slice with the largest ℓ_2-norm can be eliminated from the stack. The averaged image is then recalculated and the process is iterated until a given number of frames are removed. This is useful especially when averaging a large number of frames.

Finally, the corresponding spectrum images of the remaining frames are averaged. If the SNR is still too low to map the fine spectral features after averaging, an additional wPCA denoising step should be introduced (Supplementary Fig. 9d). In practice, the scree plot is used to estimate the number of components, which is assumed to contain all the spectral information for reconstructing the noise-free spectra. When investigating fine spectral features, however, this number cannot be determined from the scree plot, increasing the risk of introducing artifacts or removing faint spectral features at reconstruction.[24,25] An interactive selection of the number of components is implemented to check if features are only present at a specific number of components, which would indicate an artifact.

The post-processing code can be downloaded from `https://github.com/MichaelO1993/PostProcessing`.

References

(1) Xu, W.; LeBeau, J. A deep convolutional neural network to analyze position averaged convergent beam electron diffraction patterns. *Ultramicroscopy* **2018**, *188*, 59–69.

(2) Oberaigner, M.; Clausen, A.; Weber, D.; Kothleitner, G.; Dunin-Borkowski, R. E.; Knez, D. Online Thickness Determination with Position Averaged Convergent Beam

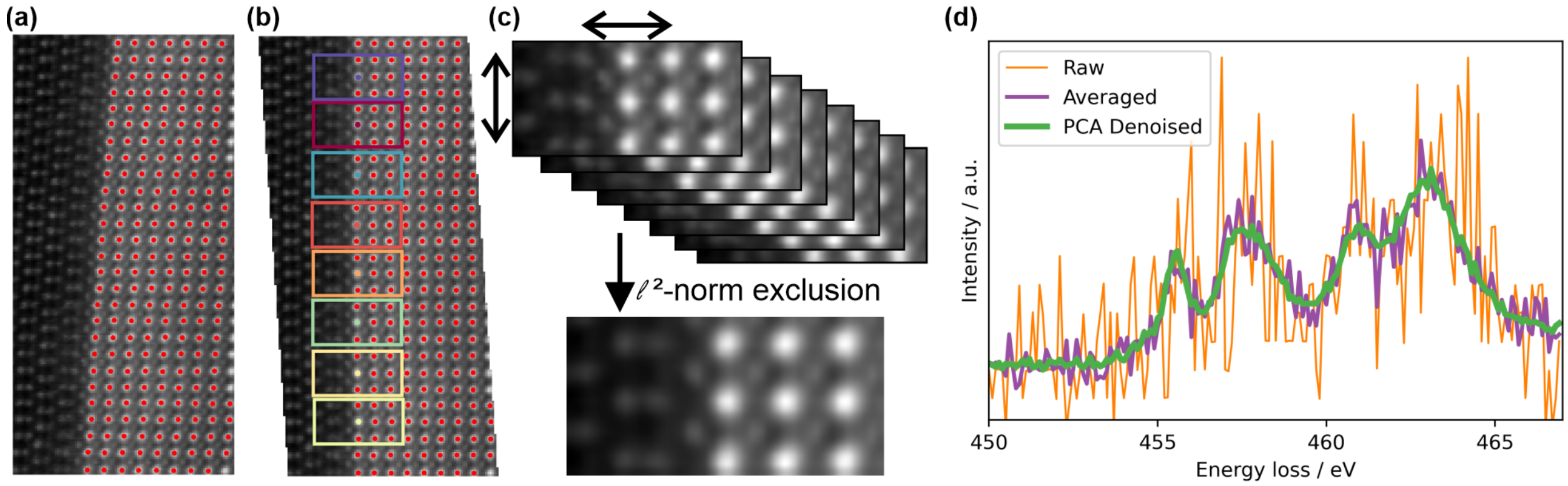

Supplementary Fig. 9: Multi-frame approach utilizing the periodicity of the crystal. (a) Determined atom positions. (b) Drift-corrected HAADF image with patches, which are cropped from the image. (c) Rigid and non-rigid alignment of the patches. The stack is averaged after it is checked by an ℓ_2-norm. (d) Comparison of single pixel spectra from the raw and averaged spectrum image. Additionally, a PCA denoising can be applied.

Electron Diffraction using Convolutional Neural Networks. *Microscopy and Microanalysis* **2023**, *29*, 427–436.

(3) Blaha, P.; Schwarz, K.; Tran, F.; Laskowski, R.; Madsen, G. K. H.; Marks, L. D. WIEN2k: An APW+lo program for calculating the properties of solids. *The Journal of Chemical Physics* **2020**, *152*, 074101.

(4) Perdew, J. P.; Burke, K.; Ernzerhof, M. Generalized Gradient Approximation Made Simple. *Phys. Rev. Lett.* **1996**, *77*, 3865–3868.

(5) Löffler, S. Study of Real Space Wave Functions with Electron Energy Loss Spectrometry. Ph.D. thesis, Vienna University of Technology, 2013.

(6) Kirkland, E. *Advanced Computing in Electron Microscopy*; Springer New York: NY, 1998.

(7) Löffler, S.; Motsch, V.; Schattschneider, P. A pure state decomposition approach of the mixed dynamic form factor for mapping atomic orbitals. *Ultramicroscopy* **2013**, *131*, 39–45.

(8) Plimpton, S. Fast Parallel Algorithms for Short-Range Molecular Dynamics. *Journal of Computational Physics* **1995**, *117*, 1–19.

(9) Joost, W. J.; Ankem, S.; Kuklja, M. M. A modified embedded atom method potential for the titanium–oxygen system. *Modelling and Simulation in Materials Science and Engineering* **2015**, *23*, 015006.

(10) Huygh, S.; Bogaerts, A.; van Duin, A. C.; Neyts, E. C. Development of a ReaxFF reactive force field for intrinsic point defects in titanium dioxide. *Computational Materials Science* **2014**, *95*, 579–591.

(11) Knez, D.; Dražić, G.; Chaluvadi, S. K.; Orgiani, P.; Fabris, S.; Panaccione, G.; Rossi, G.; Ciancio, R. Unveiling Oxygen Vacancy Superstructures in Reduced Anatase Thin Films. *Nano letters* **2020**, *20*, 6444–6451.

(12) D'Alfonso, A.; Findlay, S.; Oxley, M.; Allen, L. Volcano structure in atomic resolution core-loss images. *Ultramicroscopy* **2008**, *108*, 677–687.

(13) Egerton, R. F. *Electron energy-loss spectroscopy in the electron microscope*, third edition ed.; The language of science; Springer: New York, 2011.

(14) Bigi, C. et al. Distinct behavior of localized and delocalized carriers in anatase TiO_2 (001) during reaction with O_2. *Physical Review Materials* **2020**, *4*, 025801.

(15) Liang, Z.; Song, D.; Ge, B. Optimizing experimental parameters of integrated differential phase contrast (iDPC) for atomic resolution imaging. *Ultramicroscopy* **2023**, *246*, 113686.

(16) Ryu, J.; Kim, H.; Kim, R. M.; Kim, S.; Jo, J.; Lee, S.; Nam, K. T.; Joo, Y.-C.; Yi, G.-C.; Lee, J.; Kim, M. Dimensionality reduction and unsupervised clustering for EELS-SI. *Ultramicroscopy* **2021**, 113314.

(17) Keenan, M. R.; Kotula, P. G. Accounting for Poisson noise in the multivariate analysis of ToF-SIMS spectrum images. *Surface and Interface Analysis* **2004**, *36*, 203–212, doi: 10.1002/sia.1657.

(18) Hinton, G.; Roweis, S. Stochastic Neighbor Embedding. Proceedings of the 15th International Conference on Neural Information Processing Systems. Cambridge, MA, USA, 2002; p 857–864.

(19) Ankerst, M.; Breunig, M. M.; Kriegel, H.-P.; Sander, J. OPTICS: Ordering Points to Identify the Clustering Structure. *SIGMOD Rec.* **1999**, *28*, 49–60.

(20) Nord, M.; Vullum, P. E.; MacLaren, I.; Tybell, T.; Holmestad, R. Atomap: a new software tool for the automated analysis of atomic resolution images using two-dimensional Gaussian fitting. *Advanced Structural and Chemical Imaging* **2017**, *3*, 9.

(21) Wang, Y.; Suyolcu, Y. E.; Salzberger, U.; Hahn, K.; Srot, V.; Sigle, W.; van Aken, P. A. Correcting the linear and nonlinear distortions for atomically resolved STEM spectrum and diffraction imaging. *Microscopy* **2018**, *67*, i114–i122.

(22) Jones, L.; Yang, H.; Pennycook, T. J.; Marshall, M. S. J.; Aert, S. V.; Browning, N. D.; Castell, M. R.; Nellist, P. D. Smart Align—a new tool for robust non-rigid registration of scanning microscope data. *Advanced Structural and Chemical Imaging* **2015**, *1*, 8.

(23) Löffler, S.; Bugnet, M.; Gauquelin, N.; Lazar, S.; Assmann, E.; Held, K.; Botton, G. A.; Schattschneider, P. Real-space mapping of electronic orbitals. *Ultramicroscopy* **2017**, *177*, 26–29.

(24) Lichtert, S.; Verbeeck, J. Statistical consequences of applying a PCA noise filter on EELS spectrum images. *Ultramicroscopy* **2013**, *125*, 35–42.

(25) Cueva, P.; Hovden, R.; Mundy, J. A.; Xin, H. L.; Muller, D. A. Data Processing for

Atomic Resolution Electron Energy Loss Spectroscopy. *Microscopy and Microanalysis* **2012**, *18*, 667–675.
